# Predicting prognosis of primary pontine hemorrhage using CT image and deep learning

**DOI:** 10.1016/j.nicl.2022.103257

**Published:** 2022-11-04

**Authors:** Shuo Wang, Feng Chen, Mingyu Zhang, Xiaolin Zhao, Linghua Wen, Wenyuan Wu, Shina Wu, Zhe Li, Jie Tian, Tao Liu

**Affiliations:** a Beijing Advanced Innovation Center for Big Data-Based Precision Medicine, School of Engineering Medicine, Beihang University, Beijing, China; b Key Laboratory of Big Data-Based Precision Medicine, Ministry of Industry and Information Technology, People’s Republic of China, Beijing, China; c Department of Radiology, Hainan General Hospital (Hainan Affiliated Hospital of Hainan Medical University), Haikou, China; d Department of Neurology, Nanfang Hospital, Southern Medical University, Guangzhou, China; e Department of Radiology, Yueyang Central Hospital, Yueyang, China; f Department of Neurology, Hainan General Hospital (Hainan Affiliated Hospital of Hainan Medical University), Haikou, China; g School of Cyberspace Science and Technology, University of Science and Technology of China, Hefei, China

**Keywords:** CT, Computed tomography, PPH, Primary pontine hemorrhage, ICH, Intracerebral hemorrhage, DL, Deep learning, Primary pontine hemorrhage, Intracerebral hemorrhage, Computed tomography, Deep learning, Prognosis

## Abstract

Prognosis of primary pontine hemorrhage (PPH) is important for treatment planning and patient management. However, only few clinical factors were reported to have prognostic value to PPH. Here, we propose a deep learning (DL) model that mines high-dimensional prognostic information from computed tomography (CT) images and combines clinical factors for predicting individualized prognosis of PPH. We proposed a multi-task DL model to learn high-dimensional CT features of hematoma and perihematomal areas for predicting the risk of 30-day mortality, 90-day mortality and 90-day functional outcome of PPH simultaneously. We further explored the combination of the DL model and clinical factors by building a combined model. All the models were trained in a training cohort (n = 219) and tested in an independent testing cohort (n = 35). The DL model achieved area under the curve (AUC) of 0.886, 0.886, and 0.759 in predicting 30-day mortality, 90-day mortality and 90-day functional outcome of PPH in the independent testing cohort, which improved over the previously reported new PPH score and the clinical model. When combining the DL model and clinical factors, the combined model achieved improved performance (AUC = 0.920, 0.941, and 0.894), indicating that DL model mines CT information that complements clinical factors. Through DL visualization technique, we found that the internal structure of hematoma and its expansion to perihematomal regions are important for predicting the prognosis of PPH. This DL model provides an easy-to-use way for predicting individualized prognosis of PPH by mining high-dimensional information from CT images, and showed improvement over clinical factors and present methods.

## Highlights

• Predict multiple prognostic events of primary pontine hemorrhage using CT image.
• A multi-task deep learning model mines both hematoma and perihematomal information.
• Deep learning (DL) model achieves superior performance than present methods.
• DL found the internal texture of hematoma contains important prognostic information.

## 1 Introduction

Primary pontine hemorrhage (PPH) is the most lethal form of intracerebral hemorrhage (ICH), with mortality rates ranging widely from 30 % to 60 % (Huang et al., 2017, Qureshi et al., 2009, Schlunk and Greenberg, 2015). Additionally, the prognosis of PPH is highly variable in different patients (Takeuchi et al., 2013). Due to the poor and highly variable prognosis, individualized prognosis prediction of PPH is important for treatment planning and patient management. Previous studies reported that some clinical factors such as age, Glasgow coma scale (GCS), and manually measured computed tomography (CT) findings (e.g., infratentorial origin and hematoma volume) showed prognostic value in predicting acute mortality and long-term functional outcome in spontaneous ICH or PPH (Hemphill et al., 2001, Hemphill et al., 2009, Meguro et al., 2015, van Ginneken et al., 2018). To eliminate the effect of choosing different cut-off values in these prognostic factors, Huang et al. developed a new PPH score combining the above clinical factors and CT observations (Huang et al., 2017). Although the new PPH score demonstrated good performance than using single predictors (Chen et al., 2021, Huang et al., 2012, Meguro et al., 2015), it only used simple CT information obtained by manual observations such as hematoma volume. The internal structure of hematoma and its invasion of surrounding tissues (e.g., hemorrhage extension and growth) are also reported to be associated with prognosis of PPH (Behrouz, 2018, Morotti et al., 2016, Singh et al., 2021), but they are not quantitatively mined by the present methods. Moreover, CT as a non-invasive method to observe the complete anatomical structure of interest contains rich high-dimensional prognostic information that are difficult for eyes to sense (Bi et al., 2019, Lambin et al., 2017). Consequently, a new method that capable of mining high-dimensional features of hematoma and its surrounding tissues from CT image is important for prognosis prediction in PPH.

Deep learning (DL) as an artificial intelligence method has shown promising results in mining high-dimensional information in CT images (Wang et al., 2019, Wang et al., 2022), detecting ICH (Chilamkurthy et al., 2018, Kuo et al., 2019, Wang et al., 2021), and hematoma volumetric analysis using CT images (Dhar et al., 2020, Ironside et al., 2019, Yu et al., 2022). Through a data-driven manner, DL model can automatically learn task-specific high-dimensional features that are difficult to be sensed by eyes but contain rich prognostic information. Convolutional neural network (CNN) is the most frequently used category of DL model (Al-masni et al., 2020, Bera et al., 2022), where the basic computational units are defined as layers (e.g. convolutional layer) and they are stacked to simulate the analysis process of human brain. Benefiting from the strong feature learning ability, DL model can mine high-dimensional features related to clinical outcomes from CT images automatically.

Compared with previous studies that only used simple hematoma size information in CT image, we propose a DL model to automatically learn high-dimensional prognostic information from CT image, aiming at providing an individualized prognosis prediction method to assist treatment planning and patient management in PPH.

## 2 Material and methods

### 2.1 Study design and participants

In this study, we collected two datasets: Training cohort (n = 219) that is retrospectively collected from Hainan General Hospital between April 2016 and October 2020, and testing cohort (n = 35) that is retrospectively collected from Nanfang Hospital between March 2017 and September 2020. All the patients were consecutively collected, and satisfied the following inclusion criteria: i) PPH diagnosed by CT imaging and admitted within 24 h after symptoms onset; ii) patients aged between 18 and 80 years; iii) CT scanning was performed covering the whole intraventricular area and DICOM image were retrieved; iv) all the patients have regular follow-up for at least 90 days and performed functional score in the 90th days after treatment. Patients were excluded if: i) with end-stage malignant diseases, hemorrhage involving cerebellum, secondary to head trauma, bleeding diathesis, a cavernous hemangioma, or an arteriovenous malformation. Detailed CT scanning parameters are provided in Supplementary methods S1.

We used 30-day mortality, 90-day mortality, and 90-day functional outcome as primary end-point of this study. Functional outcome was measured by modified Rankin Scale (Swieten et al., 1988), where score between 0 and 3 was defined as good functional outcome and score between 4 and 6 was defined as poor functional outcome (Huang et al., 2017). Information on survival and functional outcome were obtained from family members through telephone interviews by a trained neurologist blinded to study data.

### 2.2 Development of the DL model for prognostic analysis of PPH

**Fig. 1:**
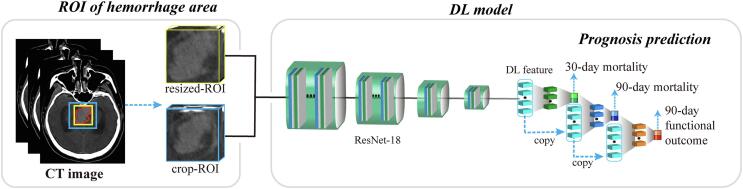
Workflow of the DL model.

Fig. 1 illustrated the pipeline of the DL model for prognostic prediction of PPH, which includes two steps: 1) region of interest (ROI) selection in CT image, and 2) DL model building.

All the CT images were firstly resampled into 0.5 × 0.5 × 5 mm using third-order spline interpolation; then, the bounding box containing the entire intraventricular hemorrhage area was manually annotated as ROI. Since the ROI size varies between different patients, we resized the ROI of different patients into the same size, which is defined as resized-ROI. However, resizing operation destroys the hemorrhage size information that is proved to be an important prognostic factor in PPH. Consequently, we introduced a crop-ROI that used a box of fixed size (90 × 90 × 36 voxels) to crop the hemorrhage area (Supplementary methods S2). Resized-ROI includes the complete hemorrhage area and is suitable for extracting detailed texture features. Crop-ROI can preserve hemorrhage size information. To combine advantages of the two ROIs, we generated both resized-ROI and crop-ROI for each patient and resized them to 90 × 90 × 36 voxels by third-order spline interpolation to construct a two-channel 3D image of size 90 × 90 × 36 × 2 (combined ROI). Afterward, we used z-score normalization to eliminate the CT intensity shift caused by different scanners. Here, we used the mean and standard deviation value of each image for z-score normalization.

After generating and standardizing the combined ROI, we used ResNet18 DL model for prognostic prediction (Supplementary Table S1), which includes four residual blocks and each residual block is multiple stacks of convolution, batch normalization, and ReLU activation layers. At the end of the last convolutional layer, we used global average pooling to generate 512-dimensional DL features. The three prognostic events in this study are associated with each other. For instance, 30-day mortality results should affect 90-day prognostic outcome. To mine the relationship between the three prognostic outcomes, 1) we used multi-task learning after extracting the DL features. Specifically, the three prognostic prediction tasks shared the 512-dimensional DL features, where each prediction task included a fully connected layer of 128 nodes and the final output layer that predicts the probability of the patient occurring the corresponding event. 2) we used the predicted 30-day mortality probability as input to the 90-day mortality prediction; and combined both the predicted 30-day mortality probability and the predicted 90-day mortality probability as input to the 90-day functional prediction, which explicitly mines the time-sequential relationship between 30-day prognostic status and 90-day prognostic status (Fig. 1). Finally, given the CT image of a patient, the DL model predicts his/her probability (DL score) of occurring 30-day mortality, 90-day mortality, and poor 90-day functional outcome, respectively.

After building the multi-task DL model, we used cross-entropy loss function and stochastic gradient descent algorithm to train the DL model (Supplementary methods S3).

### 2.3 Clinical model for predicting prognosis of PPH

To explore the prognostic value of common clinical factors, we included the 28 clinical factors shown in Table 1 and hemorrhage volume for analysis. Here, hemorrhage volume is calculated according to manually annotated hemorrhage area in CT image, which should be more precise than the simplified ABC/2 method (Huang et al., 2017, Kothari et al., 1996, Scherer et al., 2016). For very few patients that have missing value in these clinical factors, we used the mean value of the training cohort (for continuous variables) and the categorical value with the highest frequency (for categorical variables) as alternative. Afterward, all the clinical factors are normalized using z-score to achieve zero mean value and one standard deviation. For each of the three prognostic events, we used multivariate least absolute shrinkage and selection operator (LASSO) to select important clinical features that are related with the corresponding prognostic event. The hyper-parameter in LASSO is determined by threefold cross validation in the training cohort. Finally, the important clinical features selected by LASSO were used to build a logistic regression model for predicting the prognostic event. Three clinical models were therefore built for predicting 30-day mortality, 90-day mortality and 90-day functional outcome, respectively.

**Table 1:** Characteristics of patients.

	Training Cohort (n = 219)	Testing Cohort (n = 35)
Age (mean ± SD)	53.39 ± 12.29	48.5 ± 10.8
Sex (%)		
Female	50 (22.83)	2 (5.71)
Male	169 (77.17)	33(94.29)
Hypertension (%)		
Yes	168 (76.71)	23 (65.71)
No	51 (23.29)	12 (34.29)
Diabetes mellitus (%)		
Yes	16 (7.31)	3 (8.57)
No	203 (92.69)	32 (91.43)
Smoking (%)		
Former	44 (20.09)	7 (20)
Never	175 (79.91)	28 (80)
Need for mechanical ventilation (%)		
Yes	68 (31.05)	14 (0.4)
No	144 (65.75)	21 (0.6)
NA	7 (3.20)	0
CT-guided stereotactic hematoma aspiration (%)		
Yes	8 (3.65)	3 (8.57)
No	204 (93.15)	32 (91.43)
NA	7 (3.20)	0
Extraventricular drainage (%)		
Yes	19 (8.67)	4 (11.43)
No	193 (88.13)	31 (88.57)
NA	7 (3.20)	0
Alcohol abuse (%)		
Yes	51 (23.29)	8 (22.86)
No	168 (76.71)	27 (77.14)
Glucose level, median (IQR)	8.69 (6.25–8.92)	8.58 (6.3–8.88)
Temperature, °C, median (IQR)	37.5 (36.7–37.6)	37.7 (36.75–38.1)
SBP, mm Hg, median (IQR)	176.2 (154–190)	181.9 (157.5–195.5)
DBP, mm Hg, median (IQR)	101.4 (89–110)	109.7 (100–119)
MAP, mm Hg, median (IQR)	131.9 (116.5–146.7)	133.8 (123–143.3)
Heart rate, bpm, median (IQR)	93.5 (78–111)	98.2 (80–118)
Respiratory rate, bpm, median (IQR)	20.5 (18–22)	21.4 (18–23)
White cell count, 10^9^/L, median (IQR)	11.3 (7.9–14.3)	11.5 (9.3–14.0)
Hemoglobin, g/dL, median (IQR)	138.9 (130.5–153.8)	138.8 (134.5–151.2)
Platelet, 10^9^/L, median (IQR)	211.7 (171.5–249.0)	203.4 ± 41.1 (173.8–234.8)
Hematocrit, %, median (IQR)	42.3 (40.2–44.9)	42.4 (40.5–44.3)
PT, s, median (IQR)	13.1 (11.6–13.9)	13.1 (11.8–14.2)
APTT, s, median (IQR)	30.8 (26.6–34.0)	30.7 (26.4–34.0)
Urea, mmol/L, median (IQR)	6.4 (4.1–7.3)	7.1 (4.6–7.5)
Creatinine, mmol/L, median (IQR)	123.6 (66–106)	165.7 (66–109.5)
PH, median (IQR)	7.4 (7.38–7.44)	7.4 (7.38–7.44)
PaO_2_, mm Hg, median (IQR)	122.0 (87–134.7)	135.0 (94.1–179.5)
PaCO_2_, mm Hg, median (IQR)	38.8 (33.8–42.6)	40.0 (34.5–43.5)
GCS, points, median (IQR)	8.7 (6.2–8.9)	8.6 (6.3–8.88)
30-day mortality		
Alive	130 (59.36)	24 (68.57)
Dead	89 (40.64)	11 (31.43)
90-day mortality		
Alive	126 (57.53)	24 (68.57)
Dead	93 (42.47)	11 (31.43)
90-day functional outcome		
Good	116 (52.97)	27 (77.14)
Poor	103 (47.03)	8 (22.86)

Previous study built new PPH score for prognosis prediction (Huang et al., 2017), which is a combination of GCS score and hemorrhage volume based on a predefined rule. Consequently, we built the new PPH score as comparison to the DL model. As a modification to the original new PPH score, we incorporated the GCS score and hemorrhage volume into a logistic regression model to automatically learn the combination of these two factors instead of using a manually defined rule (Supplementary methods S4).

### 2.4 Combining DL model and clinical features for predicting prognosis of PPH

Previous study suggested that the combination of clinical features (e.g., GCS score) and CT information (e.g., hemorrhage volume) showed better prognostic performance. Consequently, we built a combined model integrating clinical features and the DL score to mine complementary information for better predictive performance. For predicting each prognostic outcome, we first combined all the 29 clinical factors and the DL score to form a combined feature vector, and then used LASSO to select important prognostic features from the combined feature vector. Finally, we used the selected features to build a logistic regression as the combined model for final prognostic prediction.

### 2.5 Statistical analysis

Area under the receiver operating characteristic (ROC) curve (AUC), accuracy, precision, recall, and F1-score were used to assess the performance of the models. The implementation of the DL model used the PyTorch 1.9.0 toolkit and Python 2.7.

## 3 Results

Clinical characteristics of patients in the training cohort and the testing cohort were presented in Table 1.

### 3.1 Prognostic performance of the clinical model

Among the 29 clinical factors, GCS score, hemorrhage volume, diabetes mellitus, white cell count, and need for mechanical ventilation were selected in the clinical model for predicting 30-day mortality, which achieved AUC = 0.788 in the testing cohort (Table 2). GCS score, hemorrhage volume, and need for mechanical ventilation were selected in the clinical model for predicting 90-day mortality, which achieved AUC = 0.765 in the testing cohort. GCS score, hemorrhage volume, platelet, and need for mechanical ventilation were selected in the clinical model for predicting 90-day functional outcome, which achieved AUC = 0.875 in the testing cohort. GCS score has the largest negative prognostic correlation, and hemorrhage volume has the largest positive prognostic correlation in the clinical model for predicting the three clinical outcomes (Supplementary Table S2), which is consistent with previous reports that a small GCS score and a larger hemorrhage volume usually indicate poor prognosis (Fallenius et al., 2019, Huang et al., 2017, Scherer et al., 2016, Ye et al., 2015).

**Table 2:** Prognostic performance of different models in the testing set.

		AUC (95 % CI)	Accuracy (95 % CI)	F1-score (95 % CI)	Precision (95 % CI)	Recall (95 % CI)
New PPH score	30-day mortality	0.765 (0.598, 0.906)	0.800 (0.686, 0.914)	0.632 (0.364, 0.818)	0.750 (0.500, 1.000)	0.545 (0.286, 0.800)
	90-day mortality	0.708 (0.511, 0.883)	0.771 (0.657, 0.886)	0.600 (0.333, 0.783)	0.667 (0.375, 0.917)	0.545 (0.286, 0.800)
	90-day functional outcome	0.838 (0.712, 0.946)	0.714 (0.571, 0.857)	0.545 (0.316, 0.741)	0.429 (0.222, 0.667)	0.750 (0.500, 1.000)
Clinical model	30-day mortality	0.788 (0.625, 0.932)	0.743 (0.629, 0.857)	0.609 (0.375, 0.778)	0.583 (0.333, 0.8)	0.636 (0.375, 0.875)
	90-day mortality	0.765 (0.588, 0.912)	0.743 (0.629, 0.857)	0.609 (0.370, 0.786)	0.583 (0.333, 0.818)	0.636 (0.375, 0.875)
	90-day functional outcome	0.875 (0.761, 0.966)	0.743 (0.629, 0.857)	0.609 (0.381, 0.783)	0.467 (0.250, 0.667)	0.875 (0.667, 1.000)
DL model	30-day mortality	0.886 (0.755, 0.985)	0.857 (0.743, 0.943)	0.800 (0.636, 0.923)	0.714 (0.500, 0.909)	0.909 (0.733, 1.000)
	90-day mortality	0.886 (0.744, 0.986)	0.829 (0.714, 0.914)	0.769 (0.600, 0.903)	0.667 (0.462, 0.857)	0.909 (0.733, 1.000)
	90-day functional outcome	0.759 (0.609, 0.885)	0.629 (0.514, 0.771)	0.552 (0.348, 0.720)	0.381 (0.211, 0.563)	1.000 (1.000, 1.000)
Combined model	30-day mortality	0.920 (0.833, 0.989)	0.800 (0.686, 0.914)	0.741 (0.545, 0.889)	0.625 (0.412, 0.824)	0.909 (0.750, 1.000)
	90-day mortality	0.936 (0.860, 1.000)	0.771 (0.657, 0.886)	0.733 (0.545, 0.872)	0.579 (0.375, 0.773)	1.000 (1.000, 1.000)
	90-day functional outcome	0.894 (0.791, 0.972)	0.800 (0.686, 0.914)	0.667 (0.435, 0.842)	0.538 (0.308, 0.778)	0.875 (0.667, 1.000)

Compared with the new PPH score that only included GCS score and hemorrhage volume, the clinical model included other clinical factors (e.g., diabetes mellitus, and need for mechanical ventilation) and showed better performance. As shown in Table 2, the new PPH score showed inferior performance with AUC = 0.765 in predicting 30-day mortality; AUC = 0.708 in predicting 90-day mortality; AUC = 0.838 in predicting 90-day functional outcome. The improvement of the clinical model over the new PPH score demonstrates the prognostic value of other clinical factors. This is consistent with previous studies, where diabetes mellitus (Jang et al., 2011), white cell count (Takeuchi et al., 2013), and need for mechanical ventilation (Wessels et al., 2004) were proved to be prognostic factors of PPH.

### 3.2 Prognostic performance of the DL model

The DL score revealed a significant difference between patients with good prognosis and poor prognosis in terms of 30-day mortality, 90-day mortality, and 90-day functional outcome (p < 0.0001, independent sample *t* test). We performed 10-fold cross validation in the training cohort, and the DL model achieved AUC = 0.853 in predicting 30-day mortality, AUC = 0.838 in predicting 90-day mortality, and AUC = 0.845 in predicting 90-day functional outcome (Supplementary Table S3). This performance was further confirmed in the independent testing cohort (AUC = 0.886, 0.886, 0.759, respectively, Table 2, Supplementary Fig. S1). The good performance in the testing cohort indicated that the DL model generalized well on predicting prognosis of PPH of unseen new patients.

**Fig. 2:**
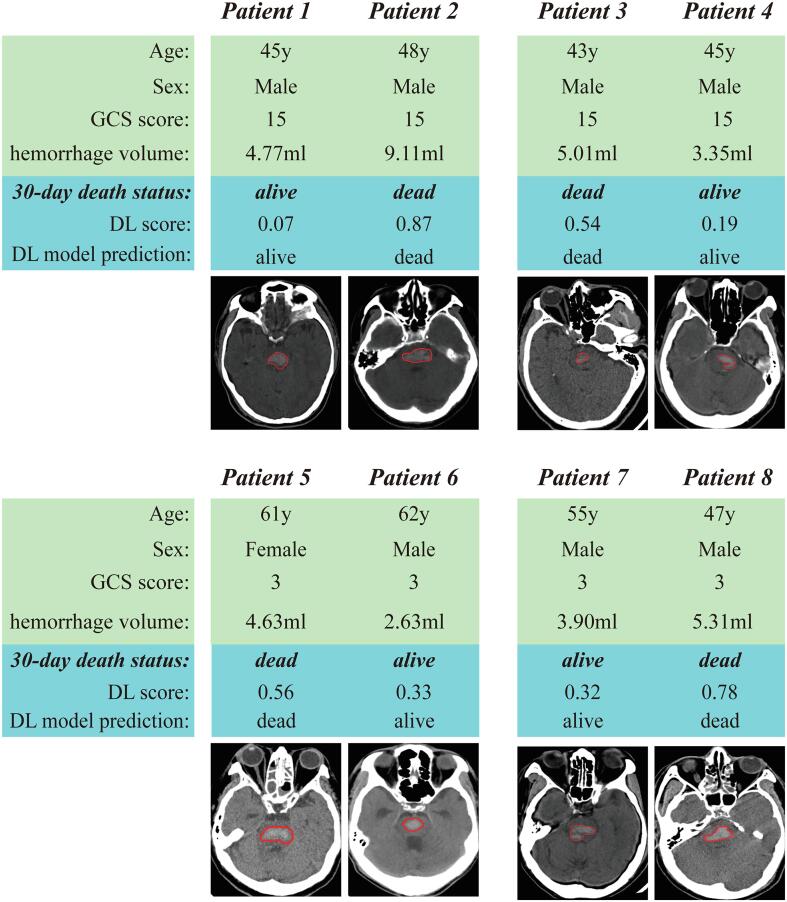
Four groups of patients with similar clinical characteristics and hemorrhage volume in CT image, but have different prognosis.

Compared with the new PPH score and the clinical model, DL model showed better prognostic value in predicting 30-day mortality and 90-day mortality, indicating that high-dimensional texture information in CT image mined by the DL model have good prognostic value, and simply use hemorrhage volume for prognostic prediction is not sufficient. For a more intuitive understanding, we illustrated four groups of patients in Fig. 2 where each group includes two patients with similar clinical characteristics (e.g., age, sex, and GCS score) and similar hemorrhage volume in CT image, but have different prognosis. Although these patients do not have large difference in clinical characteristics and hemorrhage volume, the prediction results of the DL model are discriminative, indicating that DL model is capable of mining high-dimensional prognostic information in CT image.

### 3.3 Important hemorrhage area discovered by the DL model

**Fig. 3:**
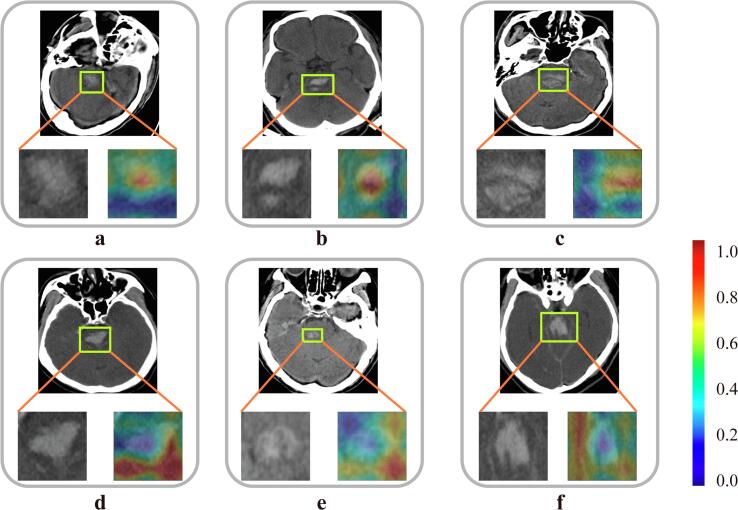
Important areas discovered by the DL model. Red color represents region with high correlation to prognosis of PPH; blue color represents region with low correlation to prognosis of PPH. (For interpretation of the references to color in this figure legend, the reader is referred to the web version of this article.)

Previous studies mainly used hemorrhage volume information from CT image; however, we found that the internal texture structure and the microenvironment around hemorrhage area are also important for predicting prognosis of PPH. Through the Grad-CAM DL visualization algorithm (Selvaraju et al., 2017), we visualized the most important area in CT image that draw attention to the DL model, which is inferred as mostly related with prognosis of PPH by the DL model. As shown in Fig. 3a, in many situations, the DL model focused on the whole hemorrhage area to learn high-dimensional prognostic information. For some patients with heterogenous intensity and obvious cracks inside hemorrhage area, the DL model focused on these specific areas (Fig. 3b, c). These structures meet the definition of previously reported blend sign, black hole sign, or swirl sign, which are defined as the hypoattenuating area encapsulated within the hyperattenuating hematoma. All these signs are associated with a poor prognosis of ICH (Li et al., 2015, Li et al., 2016, Selariu et al., 2012). Consequently, the DL model focused on hypoattenuating area inside the hyperattenuating hematoma probably because these regions may be signs of poor prognosis. Previous studies usually analyzed the inside of hemorrhage area, however, the DL model found that the microenvironment around hemorrhage area is also important for predicting prognosis of PPH (Fig. 3d–f). These results suggest that the invasion of hemorrhage area to its surrounding environment may be a prognostic sign. This finding is consistent with previous studies that hemorrhage expansion and growth is an important poor prognosis sign (Delcourt et al., 2012, Demchuk et al., 2012, Singh et al., 2021), which demonstrates the necessity of analyzing the surrounding environment of hemorrhage area. These findings suggest that the intensity distribution and internal structure of hemorrhage area, and the interactions between hemorrhage area and its surrounding environments contain important prognostic information related with PPH, which are usually ignored by previous studies relying on simple manual measurement of hemorrhage volume.

### 3.4 DL feature analysis

**Fig. 4:**
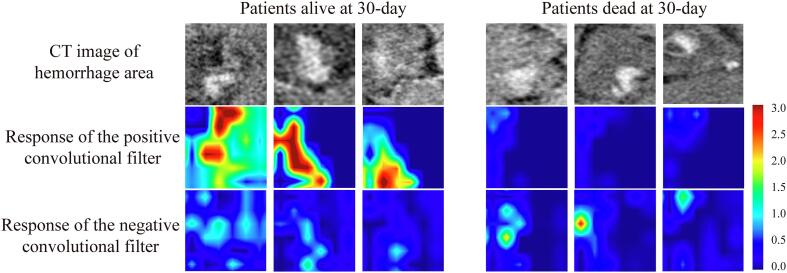
DL feature analysis. Response of the two convolutional filters to patients with different prognosis, where red color represents strong response, and blue color represents weak response. The positive filter and the negative filter are two filters from the 15th convolutional layer of the DL model. The heatmap is of 6 × 6 × 3 voxels and resized to match the input CT image (90 × 90 × 36) for display convenience. (For interpretation of the references to color in this figure legend, the reader is referred to the web version of this article.)

Compared with previous studies that only included hemorrhage volume information in CT image, DL model mines richer high-dimensional information. Benefitting from the hierarchical neural network structure, DL model can automatically learn discriminative features that have different response to patients with different prognosis. Deep convolutional layers usually contain the most discriminative features learned by the DL model, consequently, we selected two convolutional filters (defined as the positive filter and the negative filter) of the 15th convolutional layer and visualized their response to patients with different prognosis. As shown in Fig. 4, when feeding patients with good prognosis to the DL model, the positive convolutional filter has strong response, while the negative convolutional filter is nearly shut down. On the contrary, when feeding a patient with poor prognosis, the negative convolutional filter showed strong response, while the positive convolutional filter is suppressed. Consequently, the response of each convolutional filter is associated with the prognosis of the given patients, where some convolutional filters respond to patients with good prognosis while other filters respond to patients with poor prognosis. For a more intuitive observation, we extracted the 512-dimensional DL feature from the last convolutional layer of the DL model for all the patients, and plotted the patient distribution int the 512-dimensional DL feature space. As shown in the Supplementary Fig. S2, patients with good prognosis showed cluster in the DL feature space, which is separated from the cluster of patients with poor prognosis.

### 3.5 Prognostic performance of the combined model

Clinical factors and CT image provide prognostic information on micro-level biological aspect and macro-level aspects respectively. To combine the advantage of both micro-level and macro-level information, we built a combined model that integrates the clinical features and the DL score. As shown in Table 2, in the independent testing cohort, the combined model achieved AUC = 0.920, 0.936, and 0.894 in predicting 30-day mortality, 90-day mortality, and 90-day functional outcome, which is superior than both the DL model (AUC = 0.886, 0.886, and 0.759) and the clinical model (AUC = 0.788, 0.765, 0.875). The combined model yielded good calibration in both the training and the testing set in predicting the three prognostic events (p > 0.05, Hosmer-Lemeshow test, Supplementary Fig. S3). These results indicate that high-dimensional features of hemorrhage area mined by the DL model provides complementary information to clinical features, resulting in an improved prognostic performance when these two types of information are combined.

Moreover, we further explored which clinical factors complement DL score. When predicting 30-day mortality, the combined model selected DL score, GCS score, diabetes mellitus, need for mechanical ventilation, platelet, and white cell count. When predicting 90-day mortality, the combined model selected DL score, GCS score, diabetes mellitus, need for mechanical ventilation, platelet, and hypertension. When predicting 90-day functional outcome, the combined model selected DL score, GCS score, need for mechanical ventilation, and platelet. Notably, in the two combined models for predicting 30-day mortality and 90-day mortality, DL score has the largest coefficient much larger than clinical features, indicating that DL model provides more prognostic information than clinical features (Supplementary Table S4). This is consistent with results in Table 2 where the DL model showed better performance than the clinical model. Among the selected clinical features, GCS score has the largest coefficient, indicating the importance of GCS score. Through the automatic feature selection by LASSO, the combined model adaptively selected the clinical features that provides complementary information to the DL score.

## 4 Discussion

As an acute intracerebral hemorrhage with high mortality, individualized prognostic prediction of PPH is important for treatment planning and patient management. In previous studies, GCS score is proved to be the most important prognostic factor. However, the prognostic value of many other clinical factors (diabetes mellitus, white cell count, hypertension, etc.) are controversial (Jang et al., 2011, Takeuchi et al., 2013, Wessels et al., 2004). In this study, we included 29 clinical factors, and built clinical model to explore their prognostic value in predicting the three prognostic events in PPH. GCS score and hemorrhage volume as the previously reported important prognostic factors have the largest coefficients in the three clinical models. However, the clinical model also selected other clinical factors, and achieved superior performance than the previously used new PPH score that only included GCS score and hemorrhage volume. These results indicate that other clinical factors (e.g., diabetes mellitus, white cell count, platelet, and need for mechanical ventilation) also have prognostic value and can improve predictive performance of the new PPH score.

Previous studies only included few CT features such as hemorrhage volume. The internal structure of hemorrhage area and its invasion to surrounding tissues (e.g., hemorrhage extension and growth) are also reported to be associated with prognosis of PPH, but they are not quantitatively involved in the previous methods (Behrouz, 2018, Morotti et al., 2016). Through a data-driven learning manner, the DL model mines much high-dimensional CT features that can thoroughly describe the characteristics of hemorrhage area and its invasion to surrounding structures (Fig. 3). Quantitative results in Table 2 and Fig. 4 further demonstrated the effectiveness of these high-dimensional CT features mined by the DL model. Compared with extracting traditional image features in hematoma by radiomics analysis (Supplementary methods S5), the DL model also showed large improvement (Supplementary Table S5).

Given the good prognostic value of some clinical factors and the DL score, we used combined model to select clinical factors that provides information complement the DL score, and achieved superior performance than both the clinical model and the DL model. These results demonstrate the necessity of combining clinical factors and high-dimensional CT features. Previous scoring system such as the new PPH score only involved GCS score and hemorrhage volume, which only included a single clinical factor and a simple CT feature. Compared with the new PPH score, the combined model identified more prognostic factors (e.g., diabetes mellitus, need for mechanical ventilation, platelet, white cell count, and hypertension) and involved the DL score that contains richer high-dimensional CT information.

Despite the good performance of the DL model, we found some cases that are difficult for the DL model to predict. Supplementary Fig. 4a-c show images of a 30-day survivor. This patient has a low GCS score, and the hematoma presented irregular boundary, which may be a sign of hemorrhage expansion and growth. Moreover, there is a small hematoma separate from the main hematoma, which is defined as satellite sign. All these signs are usually associated with poor prognosis (Shimoda et al., 2017, Yu et al., 2017). Consequently, the DL model predicts this patient as having poor prognosis. However, this patient survived after 30 days. Patient 2 has a relatively regular boundary and small hemorrhage volume, which should have good prognosis as the DL model predicted. However, this patient died in 30 days. The unusual prognosis of these two patients may be caused by surgery results and personal constitution differences. Therefore, building a more accurate prediction model may need to incorporate postoperative information, such as including both CT images before operation and after operation for analysis. In addition, due to the relatively low incidence rate of PPH and the manual image annotation effort, the independent testing set in this study is relatively small. Consequently, the confidence intervals of some metrics of the DL model in the testing set overlap with the new PPH score. We performed 10-fold cross validation in the training set for a more thorough performance assessment, but a larger dataset especially with different populations are more desired for evaluating the proposed method in future research. We evaluated robustness of the DL model regarding imaging data from different sites and devices (Supplementary methods S1). However, different image scanning parameters may affect performance of DL models, future study can introduce new techniques such as domain adaption method to further improve performance of the DL model regarding different CT scanning parameters (Guan and Liu, 2021). Finally, our DL model relies on manually annotating hemorrhage area in CT image, future study can use an automatic hemorrhage area segmentation algorithm to acquire ROI automatically.

## 5 Conclusions

This study provides a non-invasive and easy-to-use method for predicting prognosis of PPH by combining clinical factors and commonly used CT images without adding additional costs. The performance improvement of the combined model over the previous methods further demonstrates the importance of mining high-dimensional CT features for PPH analysis.

## Declaration of Competing Interest

The authors declare that they have no known competing financial interests or personal relationships that could have appeared to influence the work reported in this paper.

## Data Availability

Data will be made available on request.
