# Supplementary material for: Predicting prognosis of primary pontine hemorrhage using CT image and deep learning
Source: Neuroimage Clin. 2022 Nov 4;36:103257. doi: 10.1016/j.nicl.2022.103257 (PMC9668666; doi:10.1016/j.nicl.2022.103257)

**Supplementary Material**

**Supplementary Methods S1.** CT scanning parameters.

***Hainan General Hospital:*** Head CT images of 219 patients were acquired using TOSHIBA Asteion, TOSHIBA Aquilion, TOSHIBA Ingenuity CT, and Philips SOMATOM Definition scanners in Hainan General Hospital. The acquisition parameters of TOSHIBA Asteion were as follows: tube voltage=120 kV; tube current=200 mA; rotation time=0.5 s; slice thickness was 4 mm or 8mm; pixel matrix=512 $\times$ 512; spiral pitch factor=0.656; reconstruction diameter=220 mm. The TOSHIBA Aquilion used the following acquisition parameters: tube voltage=120 kV; tube current=200 mA; rotation time=0.5 s; slice thickness was 4 mm or 8mm; pixel matrix=512 × 512; spiral pitch factor=0.656; Reconstruction diameter=220 mm. The TOSHIBA Ingenuity CT used the following acquisition parameters: tube voltage=120 kV; tube current=180 mA; rotation time=0.5 s; slice thickness=5 mm; pixel matrix=512×512; spiral itch factor=0.3; reconstruction diameter=250 mm. The Philips SOMATOM Definition used the following acquisition parameters: tube voltage=120 kV; tube current=240 mA; rotation time=0.5 s; slice thickness was 7.2 mm; pixel matrix=512×512; spiral pitch factor=0.9; reconstruction diameter was 238 mm.

***Nanfang Hospital:*** In Nanfang hospital, head CT images of 35 patients were acquired using the three types of CT scanners: SOMATOM Definition and SOMATOM PLUS 4 (Siemens Medical Solutions, Forchheim, Germany), and iCT 256 (Philips Medical Systems, Amsterdam, Netherlands). For the SOMATOM Definition and SOMATOM PLUS 4 scanners, scanning parameters were as follows: tube voltage=120 kV; tube current was 240 mA or 630mA; spiral pitch factor of 0.9; rotation time=0.4 s; slice thickness was 1.2-7.2 mm; reconstruction diameter was 226-375 mm; pixel matrix=512 $\times$ 512. For the iCT 256 scanner, scanning parameters were as follows: tube voltage=120 kV; tube current was 333 mA; rotation time=0.4 s; slice thickness was 1.0-5.0 mm; reconstruction diameter was 217-250 mm; pixel matrix=512 $\times$ 512.

**Supplementary Methods S2.** Details of acquiring ROI

Two ROIs were used in this study: resized-ROI and crop-ROI. When acquiring resized-ROI, we first used a bounding box (defined as ROI) to cover the entire intraventricular hemorrhage area. The size of this ROI varies between each patient, consequently, we resized this ROI to 90×90×36 voxels for each patient.

When acquiring crop-ROI, we first calculated the center coordinate of the resized-ROI, then, used a cube of fixed size (90×90×36 voxels) located at the center coordinate to crop the image. During the crop operation, there are two special situations: 1) If the crop-ROI exceeds CT image boundary, we shift it to align to CT image boundary. For instance, if the coordinate of a crop-ROI starts at 432 and ends at 432+90=522, but the CT image matrix is 512 in this dimension, then, we shift the crop-ROI by 10 voxels left to ensure it align to the boundary of the CT image, finally, the coordinate of the crop-ROI starts at 432-10=422, and ends at 422+90=512. 2) if the hematoma is larger than 90×90×36 voxels, we only reserve the center part (90×90×36 voxels) of the hematoma.

All the ROIs are normalized through z-score normalization to eliminate the CT intensity shift caused by different scanners. Suppose $x_{old}$ be the value of a voxel in CT image, $x_{mean}$ be the mean value of the CT image, $x_{std}$ be the standard deviation of the CT image. Then, the voxel value is normalized by $x_{new}=\frac{x_{old}-x_{mean}}{x_{std}}$. Through this normalization, all the ROIs have zero mean value and one standard deviation, which can eliminate the image intensity bias caused by different equipment.

**Supplementary Methods S3.** Training process of the DL model

Model training aims at optimizing the parameters of the DL model to build the relationship between CT image and prognosis of PPH. The model training is an iterative process, which optimizes the model at each iteration until the model achieves the best predictive performance. Although we have three prognostic end-points, we adopted three binary prediction tasks instead of a categorical prediction task because binary prediction task is relatively easier than multi-class categorical prediction task, especially when the training data amount is not big. For predicting 30-day mortality and 90-day mortality, we used 1 to represent alive, and 0 to represent dead. For predicting 90-day functional outcome, we used 1 to represent poor functional outcome and 0 to represent good functional outcome. At each iteration, we used cross entropy as cost function to measure the predictive performance of the DL model:

$$L\left( w \right)=\frac{1}{N}\sum_{n=1}^{N} \left[ y_{n}logp_{n}+\left( 1-y_{n} \right)log\left( 1-y_{n} \right) \right]$$

In this formula, *w* is the parameter of the DL model that needs to be trained; *N* is the training sample number; $y_{n}$ represents the true prognostic status (0 for alive or good 90-day functional outcome, 1 for dead or poor 90-day functional outcome); $p_{n}$ is the predicted probability of occurring end-event. If the cost function $L\left( w \right)$ is not minimum, we use stochastic gradient descent (SGD) algorithm to update the parameters of the DL model and minimize the loss function. The learning rate of SGD is set to 0.005 and is reduced by 0.5 times when the loss stopped decreasing for 2 epochs.

We randomly selected ten percent of the training cohort (n=219) as the internal validation set (n=22) for tuning hyper-parameter (the training epoch number) of the DL model. The dataset collected from Nanfang Hospital is used as testing set. During training process, we validated performance of the DL model after each training epoch ends. In the following figure, we plotted the AUC changing curves in the internal validation set as the training epoch increases:


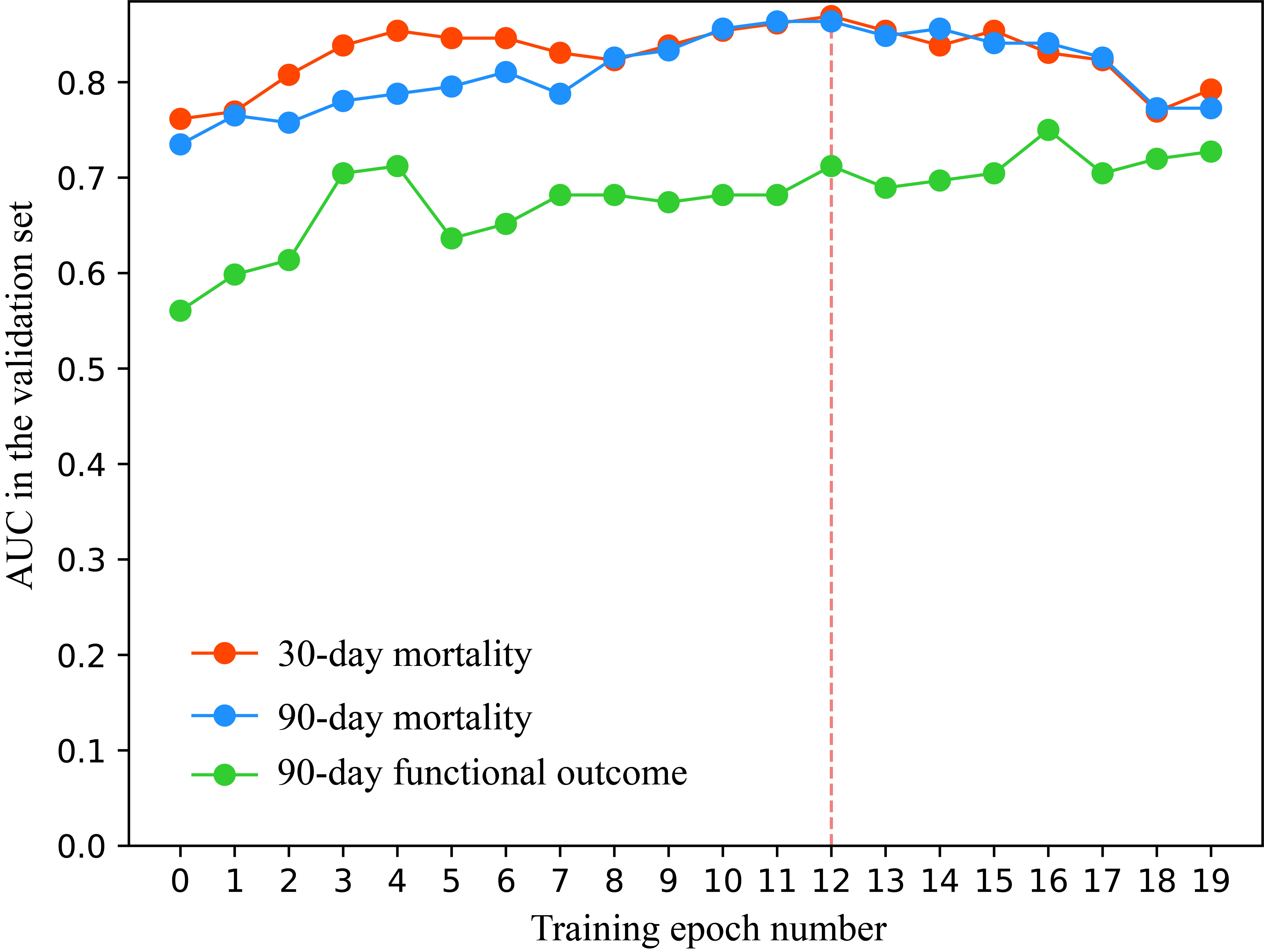


As shown in the above figure, the DL model reaches the highest AUC value at the 12^th^ epoch in terms of predicting 30-day mortality and 90-day mortality. Consequently, we finally selected the DL model trained for 12 epochs and tested its performance in the testing set.

**Supplementary Methods S4.** Details of building the new PPH score

The original new PPH score is calculated according to a pre-defined scoring rule:

| **Variables** | **New PPH score point** |
| --- | --- |
| GCS score |  |
| 3-4 | 2 |
| 5-7 | 1 |
| 8-15 | 0 |
| PPH volume |  |
| >10ml | 2 |
| 5-10 ml | 1 |
| < 5ml | 0 |

For each patient, a new PPH score is calculated according to his/her GCS score and PPH volume according to the above scoring rule, which can be represented as $y=GCS\_score+PPH\_volume$, and the predicted value y is converted to binary by using 1.5 as cut-off value.

In this study, as a modification to the original new PPH score, we incorporated the GCS score and hemorrhage volume into a logistic regression model to automatically learn the combination of these two factors instead of using a manually defined rule. Namely, the new PPH score in this study can be represented as $y=w_{1}*GCS\_score+w_{2}*PPH\_volume$, where $w_{1}$ and $w_{2}$ are parameters learned by logistic regression model automatically.

**Supplementary Methods S5.** Radiomics model building process

After acquiring the ROI of hematoma in CT image by manual annotation, we used PyRadiomics toolkit to extract 1051 traditional image features, including 111 features extracted from the original ROI image, 188 LoG texture features, and 752 wavelet features.

| **Feature type** | **Feature name** | **Feature number** |
| --- | --- | --- |
| Original image feature |  |  |
|  | First Order Statistics | 19 |
|  | Shape-based (3D) | 17 |
|  | Gray Level Cooccurence Matrix | 24 |
|  | Gray Level Run Length Matrix | 16 |
|  | Gray Level Size Zone Matrix | 16 |
|  | Neighbouring Gray Tone Difference Matrix | 5 |
|  | Gray Level Dependence Matrix | 14 |
| texture | LoG | 188 |
| Wavelet | Wavelet | 752 |

Afterward, we used LASSO to select important features associated with the prognosis of PPH. Finally, support vector machine (SVM) was used to predict the prognosis of PPH. The parameters of LASSO and SVM are determined by 5-fold cross validation in the training set. Since there are three prognostic end-points, the feature selection and model building process were performed individually for each end-point.

Finally, the radiomics features selected for predicting the three prognostic end-points are as follows:

|  | **Name of the selected radiomics features** |
| --- | --- |
| 30-day mortality prediction | log-sigma-3-mm-3D_glrlm_LongRunHighGrayLevelEmphasis  wavelet-HLL_glszm_SmallAreaLowGrayLevelEmphasis  wavelet-HHH_glcm_SumSquares  wavelet-HLH_firstorder_Kurtosis  log-sigma-5-mm-3D_firstorder_Kurtosis  wavelet-LHL_glrlm_RunEntropy |
| 90-day mortality prediction | wavelet-HHH_glcm_MCC  log-sigma-3-mm-3D_firstorder_Kurtosis  log-sigma-3-mm-3D_glcm_Idmn  wavelet-HLH_firstorder_Kurtosis  wavelet-LHL_glcm_Correlation  wavelet-HHH_firstorder_InterquartileRange  original_glrlm_GrayLevelNonUniformity  wavelet-HHH_glcm_SumSquares |
| 90-day functional outcome prediction | wavelet-LHH_glszm_GrayLevelNonUniformity  wavelet-HLH_firstorder_Kurtosis  wavelet-LHH_glcm_SumEntropy  wavelet-HHL_glrlm_RunVariance  log-sigma-5-mm-3D_firstorder_Skewness  wavelet-LLL_glrlm_RunEntropy  wavelet-LLL_glcm_ClusterProminence  wavelet-HHH_glcm_MCC |

**Supplementary Table S1.** Main parameters and structure of the DL model

| **Layer** | **Output size** | **Parameter** |
| --- | --- | --- |
| Input | 90×90×36×2 |  |
| Convolution | 45×45×18@64 | Filter = 64, kernel = 7×7×7, stride = 2×2×2 |
| Max pooling | 23×23×9@64 | Window=3, stride=2 |
| ResBlock1 | 23×23×9@64 | Conv: filter=64, kernel=3, stride=1  Conv: filter=64, kernel=3, stride=1,  Add  Conv: filter=64, kernel=3, stride=1  Conv: filter=64, kernel=3, stride=1,  Add |
| ResBlock2 | 12×12×5@128 | Conv: filter=128, kernel=3, stride=2  Conv: filter=128, kernel=3, stride=1  Identity Conv: filter=128, kernel=1, stride=2,  Add  Conv: filter=128, kernel=3, stride=1  Conv: filter=128, kernel=3, stride=1,  Add |
| ResBlock3 | 6×6×3@256 | Conv: filter=256, kernel=3, stride=2  Conv: filter=256, kernel=3, stride=1  Identity Conv: filter=256, kernel=1, stride=2,  Add  Conv: filter=256, kernel=3, stride=1  Conv: filter=256, kernel=1, stride=1,  Add |
| ResBlock4 | 3×3×2@512 | Conv: filter=512, kernel=3, stride=2  Conv: filter=512, kernel=3, stride=1  Identity Conv: filter=512, kernel=1, stride=2, Add |
| Global average pooling | 1×1×1@512 | Output size: (1, 1, 1) |
| Flatten1 | 512 |  |
| Classifier1 | 2 | Fully connection: input 512, output 128  Fully connection: input 128, output 2 |
| Concatenation2 | 514 | Concatenate Flatten1 and Classifier 1 |
| Classifier2 | 2 | Fully connection: input 514, output 128  Fully connection: input 128, output 2 |
| Concatenation3 | 516 | Concatenate Flatten1, Classifier 1, and Classifier 2 |
| Classifier3 | 2 | Fully connection: input 516, output 128  Fully connection: input 128, output 2 |

**Supplementary Table S2.** Coefficient of features in the clinical model

| **Clinical model** | **Feature name** | **Coefficient** |
| --- | --- | --- |
| 30-day mortality prediction | GCS score | -0.68711171 |
|  | Hemorrhage volume | 1.47179886 |
|  | Diabetes mellitus | -0.33199247 |
|  | White cell count | 0.34805609 |
|  | Need for mechanical ventilation | 0.28528233 |
| 90-day mortality prediction | GCS score | -0.77383003 |
|  | Hemorrhage volume | 1.39345467 |
|  | Need for mechanical ventilation | 0.3485928 |
| 90-day functional outcome prediction | GCS score | -1.43240223 |
|  | Hemorrhage volume | 1.45107706 |
|  | Platelet | 0.21833967 |
|  | Need for mechanical ventilation | 0.48105655 |

**Supplementary Table S3.** Performance of 10-fold cross validation of the DL model in the training set.

|  |  | **AUC** | **Accuracy** | **F1-score** | **Precision** | **Recall** |
| --- | --- | --- | --- | --- | --- | --- |
| 10-fold cross validation of the DL model in the training set | 30-day mortality | 0.853±0.075 | 0.807±0.085 | 0.749±0.117 | 0.828±0.165 | 0.723±0.163 |
|  | 90-day mortality | 0.838±0.083 | 0.765±0.130 | 0.770±0.097 | 0.700±0.151 | 0.891±0.112 |
|  | 90-day functional outcome | 0.845±0.110 | 0.776±0.140 | 0.805±0.092 | 0.741±0.168 | 0.921±0.103 |

Note: The results in this table is presented as mean ± std of the ten validation set in the 10-fold cross validation.

**Supplementary Table S4.** Coefficient of features in the combined model.

| **Combined model** | **Feature name** | **Coefficient** |
| --- | --- | --- |
| 30-day mortality prediction | DL score | 1.8585549 |
|  | GCS score | -0.6022602 |
|  | Diabetes mellitus | -0.55484704 |
|  | white cell count | 0.39980656 |
|  | platelet | 0.33588482 |
|  | Need for mechanical ventilation | 0.23271192 |
| 90-day mortality prediction | DL score | 1.77924736 |
|  | GCS score | -0.69424933 |
|  | hypertension | -0.35483888 |
|  | Diabetes mellitus | -0.40034099 |
|  | platelet | 0.35401347 |
|  | Need for mechanical ventilation | 0.32145744 |
| 90-day functional outcome prediction | DL score | 1.64835421 |
|  | GCS score | -1.38723499 |
|  | platelet | 0.30829513 |
|  | Need for mechanical ventilation | 0.41331434 |

**Supplementary Table S5.** Prognostic performance of the radiomics model

|  |  | **AUC** **(95% CI)** | **Accuracy (95% CI)** | **F1-score (95% CI)** | **Precision (95% CI)** | **Recall (95% CI)** |
| --- | --- | --- | --- | --- | --- | --- |
| Radiomics model | 30-day mortality | 0.739 (0.577, 0.883) | 0.743 (0.629, 0.857) | 0.609 (0.364, 0.788) | 0.583 (0.333, 0.833) | 0.636 (0.375, 0.875) |
|  | 90-day mortality | 0.735 (0.565, 0.876) | 0.686 (0.543, 0.829) | 0.476 (0.200, 0.690) | 0.500 (0.200, 0.778) | 0.455 (0.182, 0.714) |
|  | 90-day functional outcome | 0.720 (0.564, 0.872) | 0.686 (0.543, 0.800) | 0.522 (0.286, 0.720) | 0.500 (0.250, 0.750) | 0.545 (0.286, 0.800) |

**Supplementary Fig. S1.** Receiver operating characteristic (ROC) curves


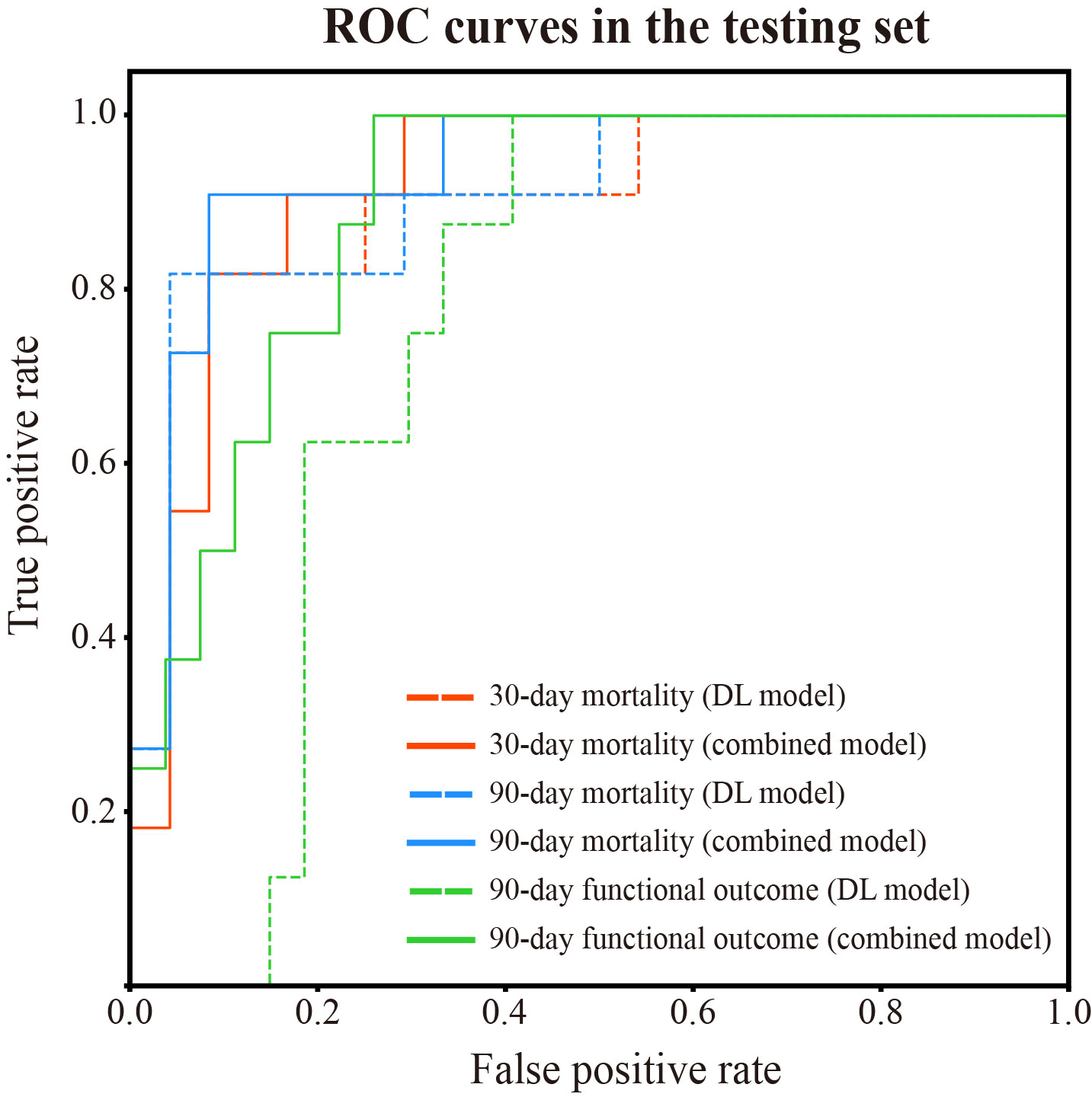


**Supplementary Fig. S2.** Patient distribution in the 512-dimensional DL feature space. Principal component analysis is used to reduce the 512-dimensional feature space into 2-dimensional space for display convenience (implemented by KernelPCA function in the *sklearn* toolkit with the following default parameter settings: n_components = 2, kernel = rbf, gamma = 1/512, degree=3, and alpha=1). The first two principal components covered 57.83% and 15.38% variance.


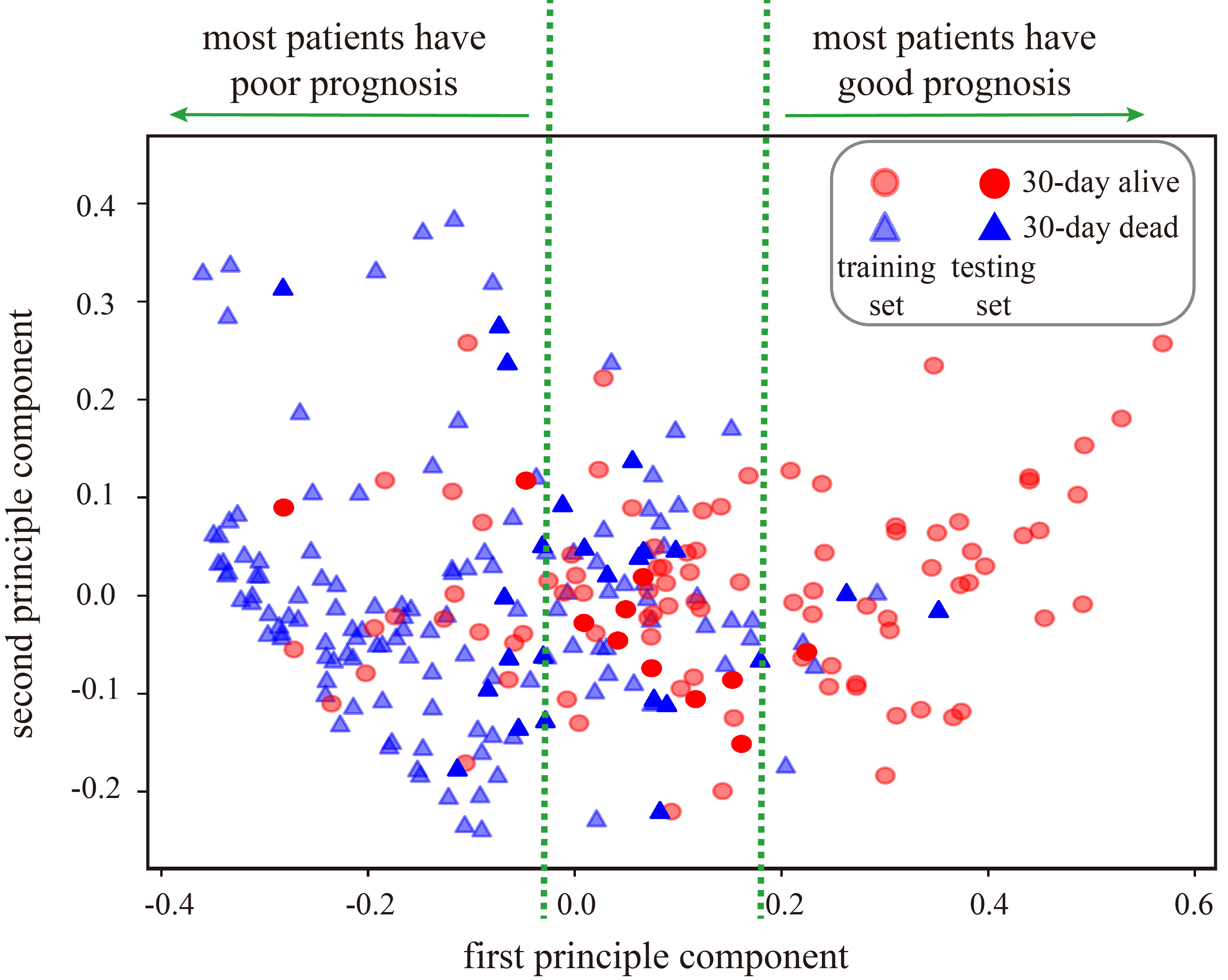


**Supplementary Fig. S3.** Calibration curves of the combined model. P-value in this figure is assessed using Hosmer-Lemeshow test. BS represents Brier score.


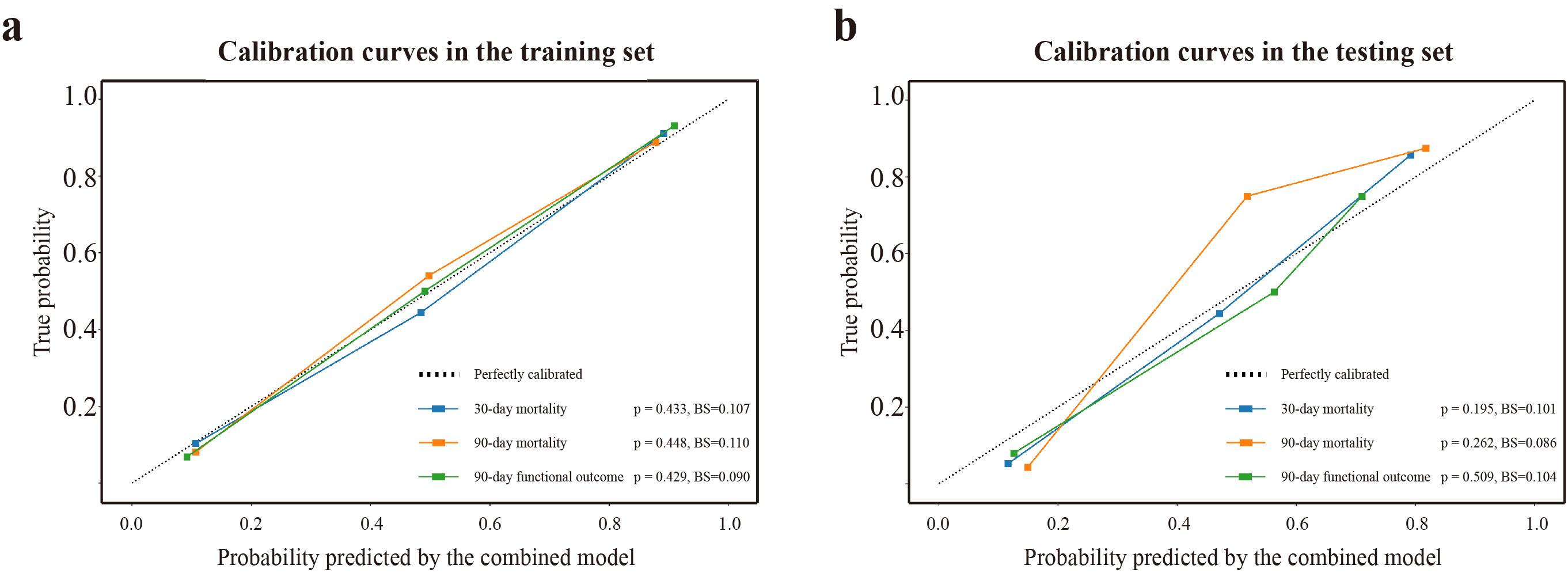


**Supplementary Fig. S4.** Two patients that the DL model generates wrong predictions


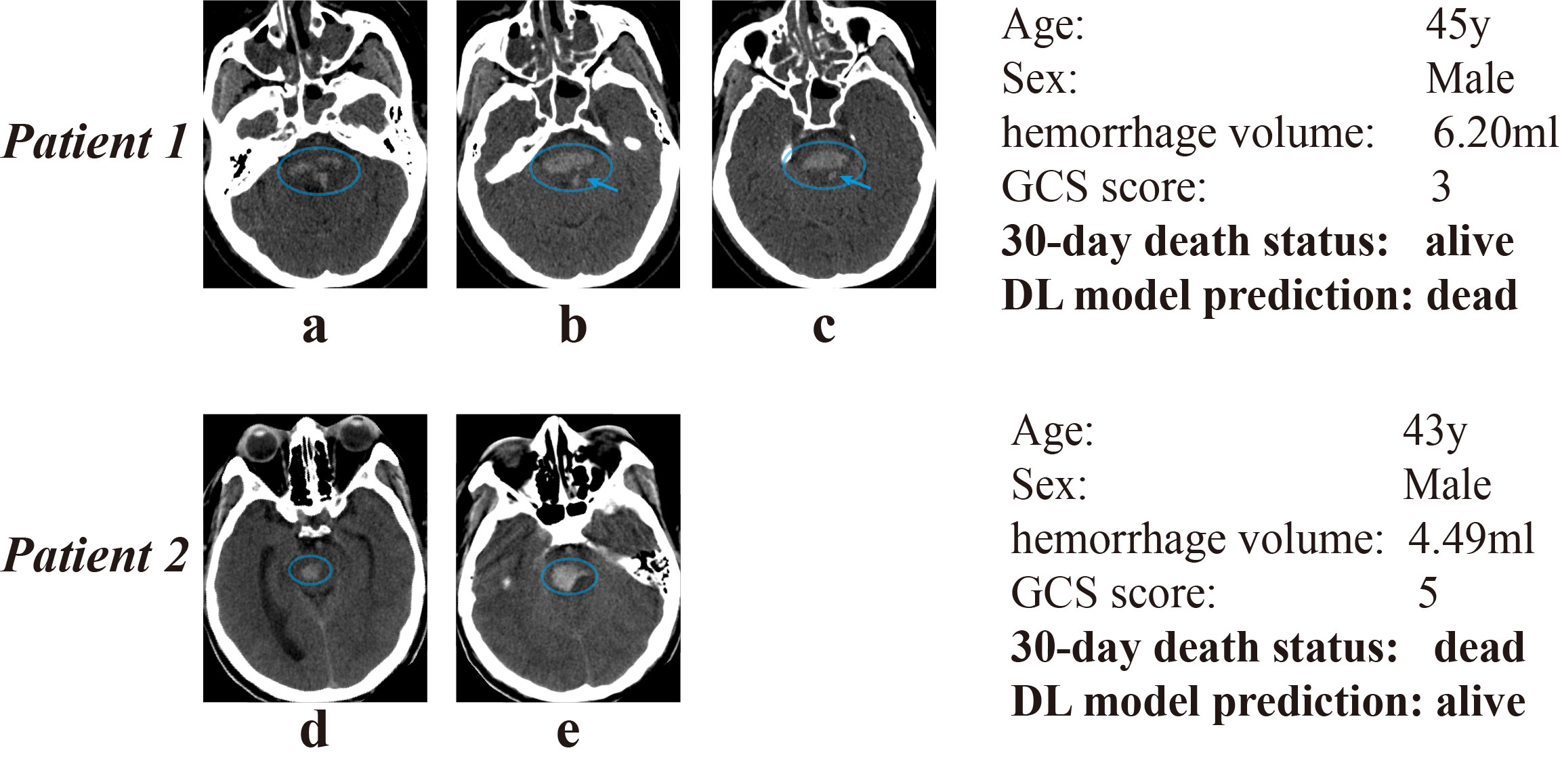

Supplement: Supplementary data 1 [file mmc1.docx]
